# Supplementary material for: DFT Study of Regio- and Stereoselective 13DC Reaction between Diazopropane and Substituted Chalcone Derivatives: Molecular Docking of Novel Pyrazole Derivatives as Anti-Alzheimer’s Agents
Source: Molecules. 2023 Feb 16;28(4):1899. doi: 10.3390/molecules28041899 (PMC9964806; doi:10.3390/molecules28041899)
Supplement: Supplementary file 1 [file molecules-28-01899-s001.zip › molecules-2205941-supplementary.pdf]

DFT study of regio- and stereoselective [3+2] cycloaddition between diazopropane and substituted chalcone derivatives and molecular docking of novel pyrazole derivatives as anti-Alzheimer's agents

Sadeq M. Al-Hazmy [*1], Mohamed Oussama Zouaghi[2], Youssef Arfaoui[2], Waleed Koko[3], Younes Bouazizi[4] and Naceur Hamdi[*5]

[1] Chemistry Department, College of Science, Qassim University, Buraidah 51452, Saudi Arabia
[2] Laboratory of Characterizations, Applications and Modeling of Materials (LR18ES08), Department of Chemistry, Faculty of Sciences, University of Tunis El Manar, 2092 Tunis-Tunisia.
[3]Department of Science Laboratories, College of Science and Arts, Qassim University, Ar Rass 52719, Saudi Arabia
[4]Chemistry Department, Faculty of Science, Jazan University, 2097, Saudi Arabia
[5]Department of Chemistry, College of Science and Arts, Qassim University, Ar Rass 52719, Saudi Arabia
* Correspondence: hamdi_naceur@yahoo.fr, 141301@qu.edu.sa

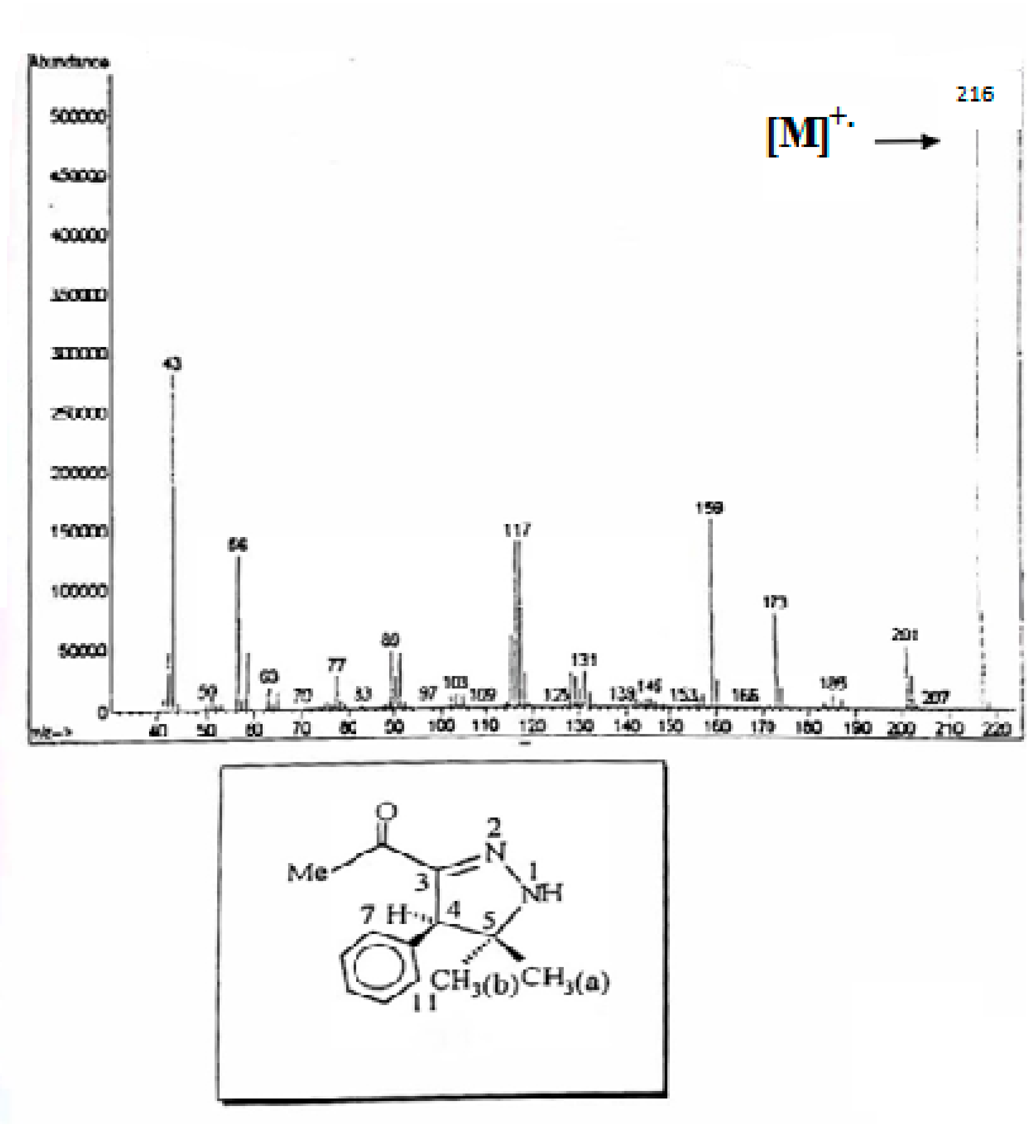

Figure S1. Mass spectrum in EI mode of D2 compound

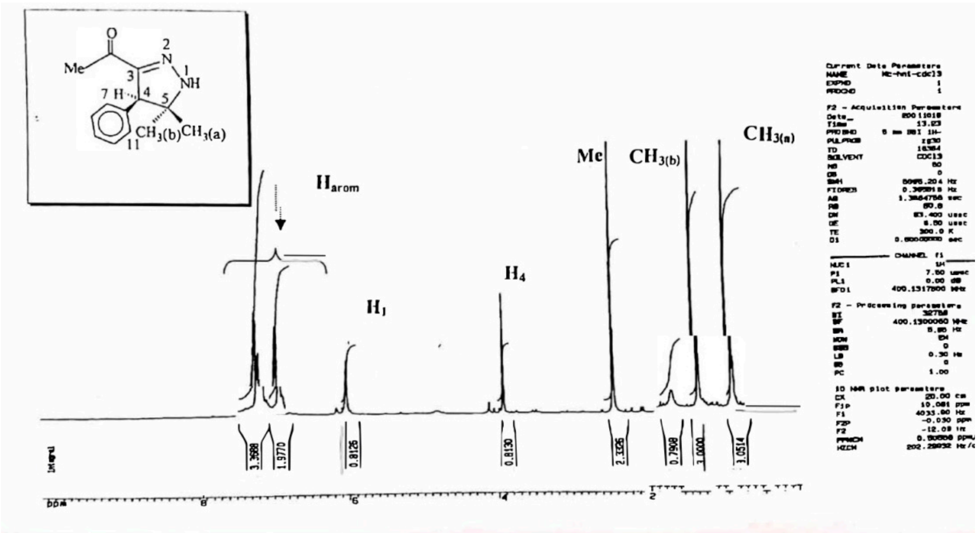

Figure S2. ^{1}H NMR spectrum((CDCl3, 300MHz) of D2 compound

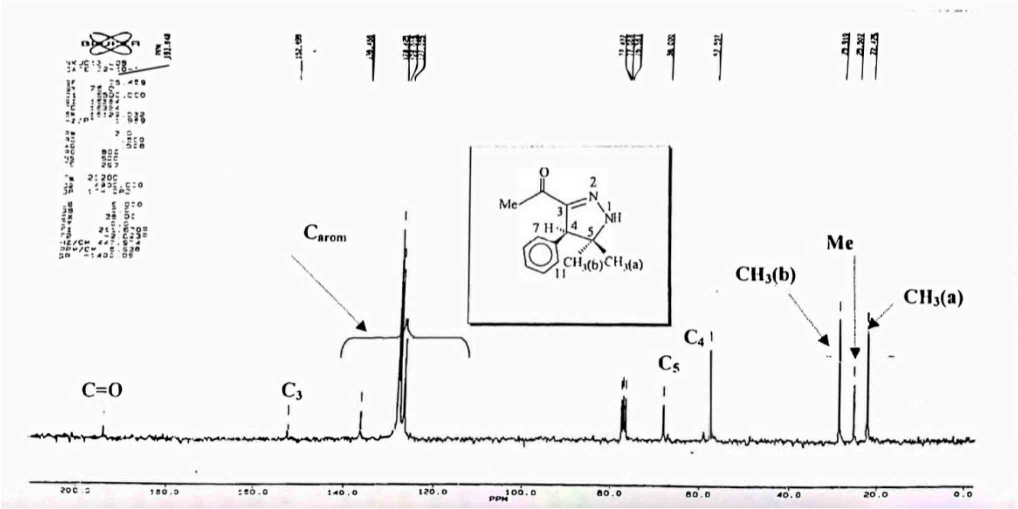

Figure S3. ^{13}C NMR spectra (CDCl3, 75MHz) of D2 compound

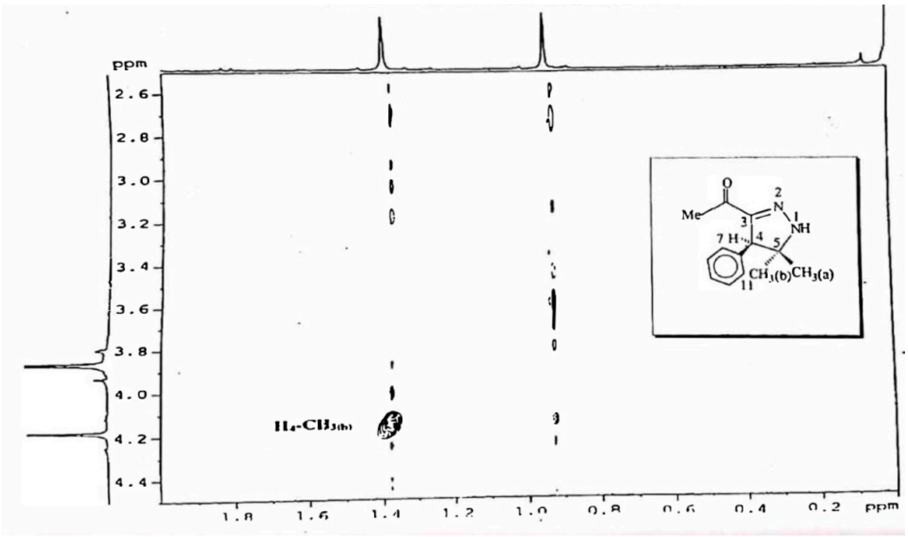

Figure S4. ^{1}H NMR-NOESY spectrum of D2 compound

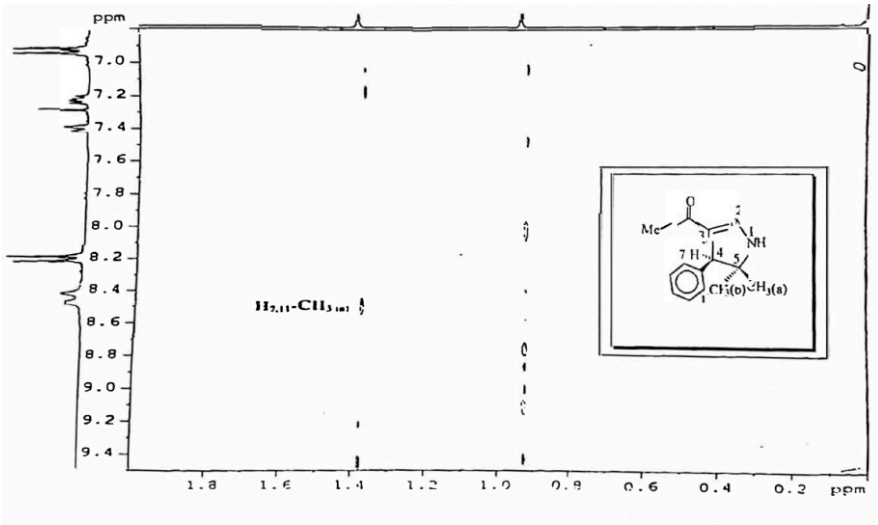

Figure S5. ^{1}H NMR-NOESY spectrum of D2 compound (0.2 - 1.8 ppm)

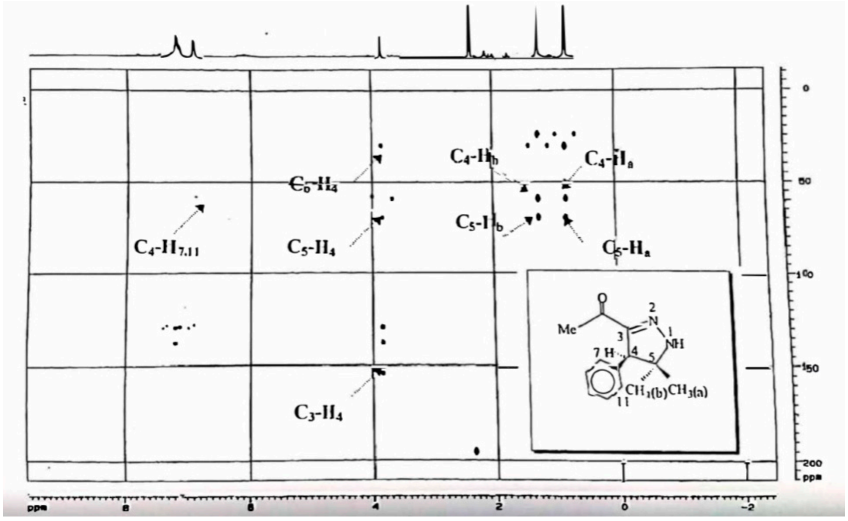

Figure S6. HMBC spectrum of D2 compound

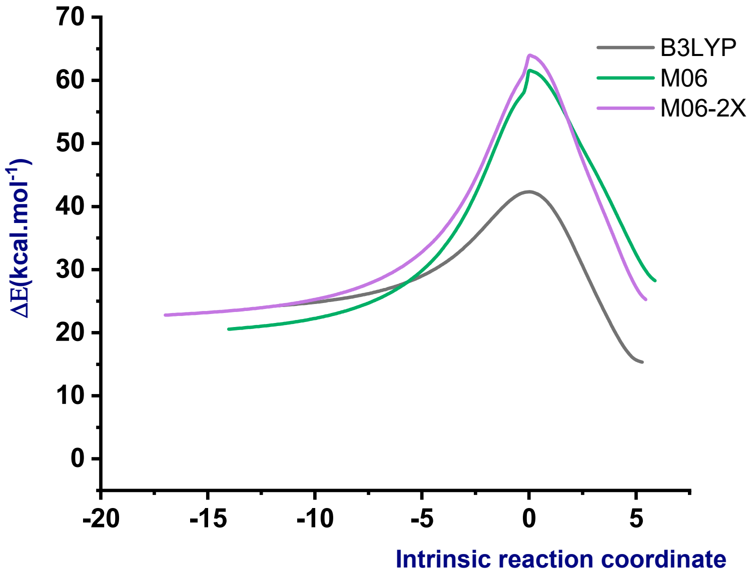

Figure S7. IRC plots of 1HP1 at B3LYP, M06 and M06-2X functional with 6-311+G(d, p) basis set.

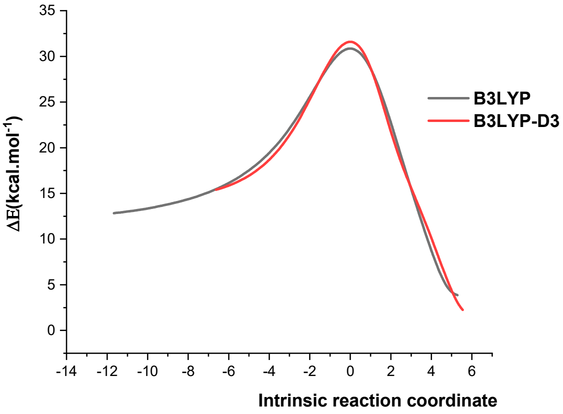

Figure S8. Computed IRC plots of the synthesis of 1HP1 at B3LYP and B3LYP-D3 functionals with 6-311+G(d, p) basis set

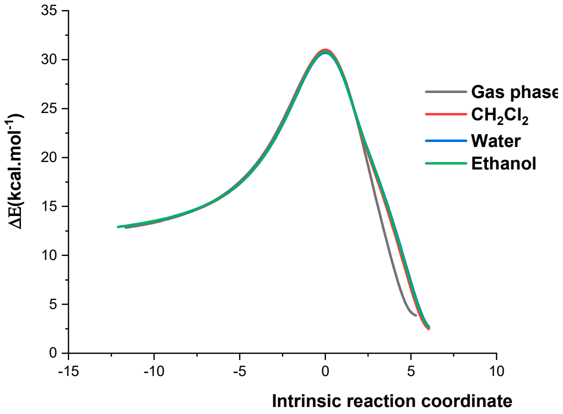

Figure S9. Computed IRC plots of the synthesis of 1HP1 at B3LYP/6-311+G(d, p) level with CPCM solvatation model in three solvents: CH_2Cl_2, water and ethanol.

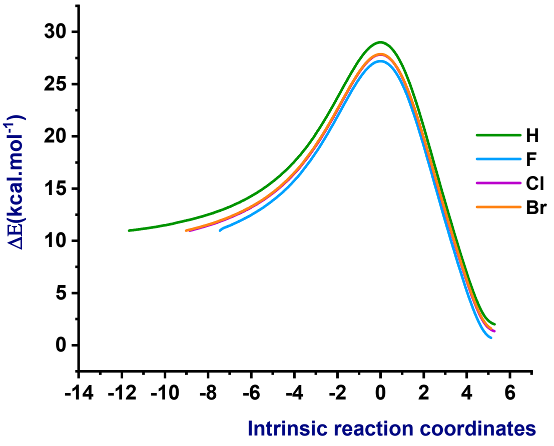

Figure S10. IRC plots as function of halogens at B3LYP level

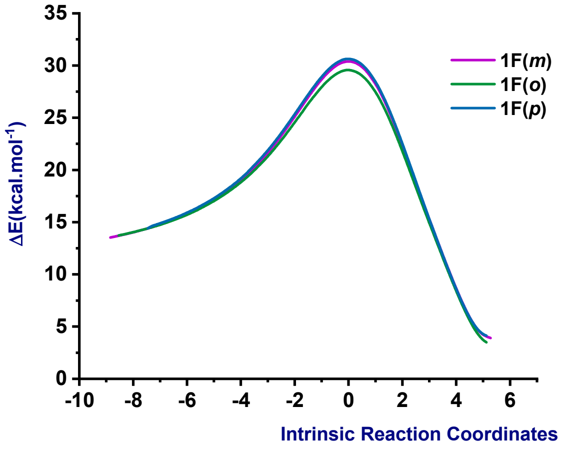

Figure S11. IRC plots of 1F(meta. ortho and para) pathways at B3LYP level

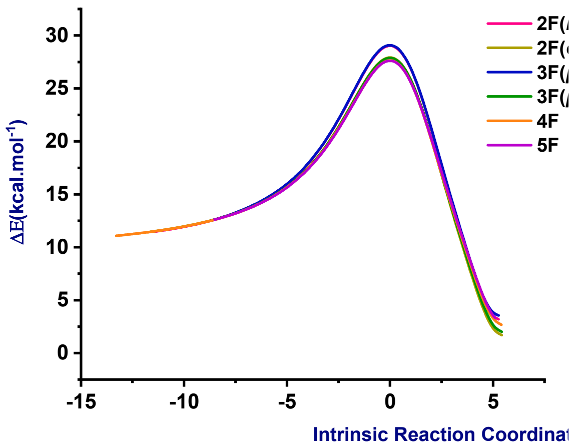

Figure S12. IRC plots of 2F. 3F. 4F and 5F pathways at B3LYP level

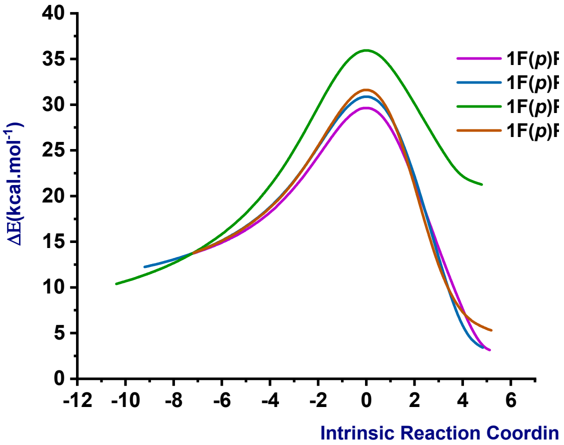

Figure S13. IRC plots of 1F(para) pathways at B3LYP level

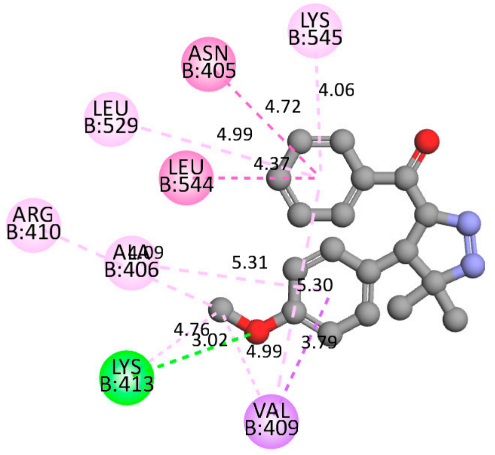

2D depiction of docking of ligand 1 in the active site of HSA (-8.3kcal/mol)

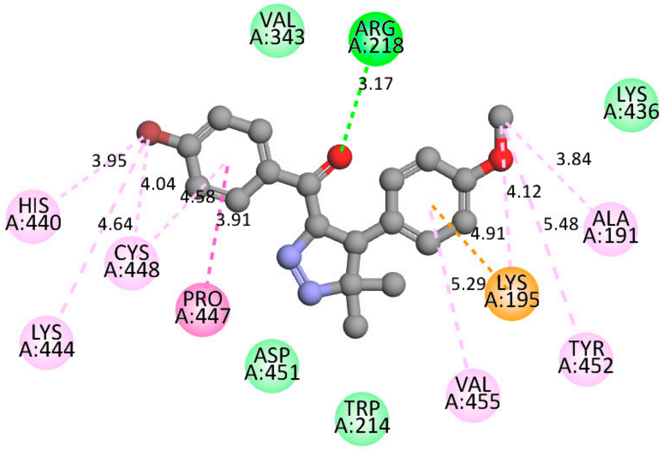

2D depiction of docking of ligand 2 in the active site of HSA (-8.6kcal/mol)

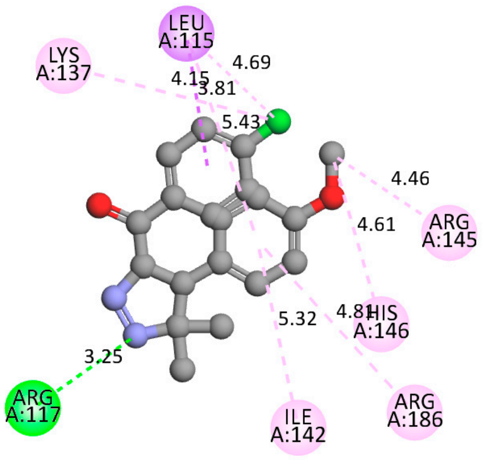

2D depiction of docking of ligand 3 in the active site of HSA (-7.8kcal/mol)

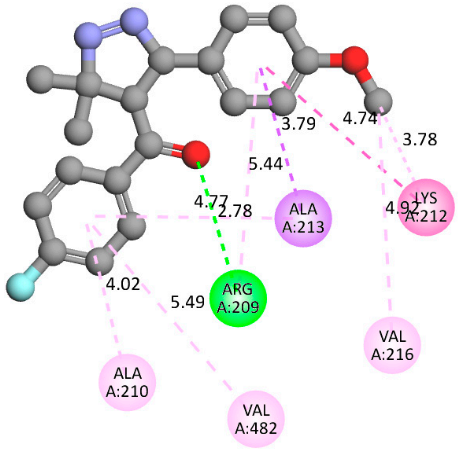

2D depiction of docking of ligand 4 in the active site of HSA (-8.5kcal/mol)

Interactions

Conventioanl Hydrogen Bond | Carbon Hydrogen Bond | Pi-Sigma | Amide-Pi Stacked | Pi-Cation | Alkyl, Pi-Alkyl

Figure S14. 2D depiction of docking of ligands 1HP1-4 in the active site of Human Serum Albumin

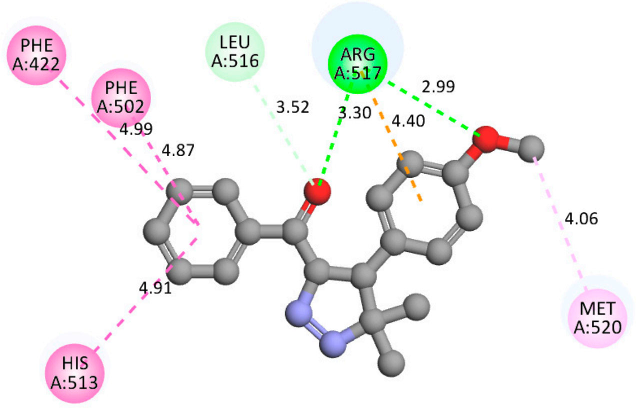

2D depiction of docking of ligand 1 in the active site of AChE

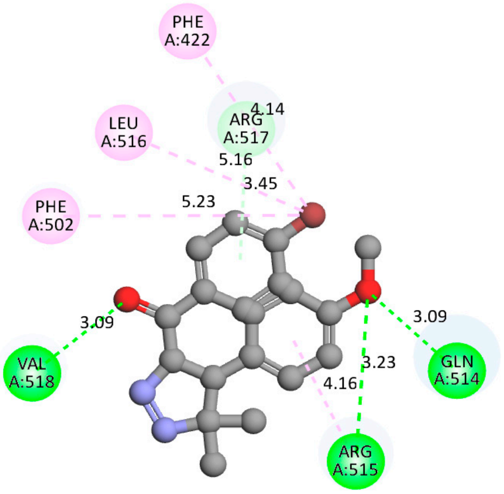

2D depiction of docking of ligand 2 in the active site of AChE

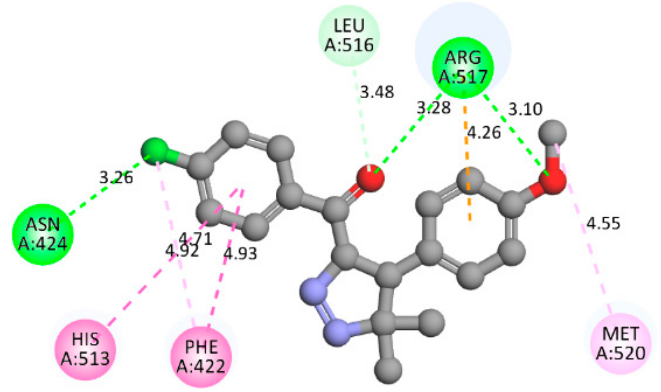

2D depiction of docking of ligand 3 in the active site of AChE

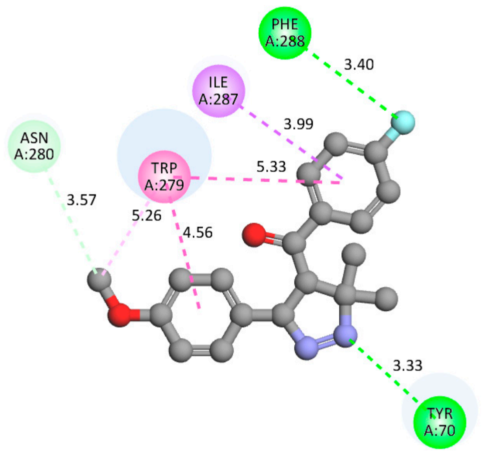

2D depiction of docking of ligand 4 in the active site of AChE

Interactions

Conventioanl Hydrogen Bond | Carbon Hydrogen Bond | Pi-Donor Hydrogen | Halogen (Fluorine)
Alkyl, Pi-Alkyl | Pi-Pi Stacked, Pi-Pi T Shaped, Amide-Pi Stacked | Pi-Cation, Pi-Anion | Pi-Sigma

Figure S15. 2D depiction of docking of ligands 1HP1-4 in the active site of AChE (PDB ID: 1ACJ)

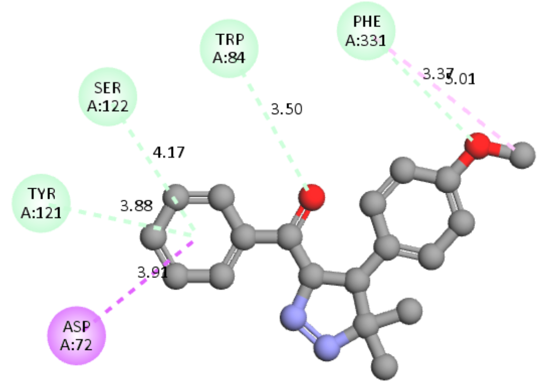

2D depiction of docking of ligand 1 in the active site of AChE

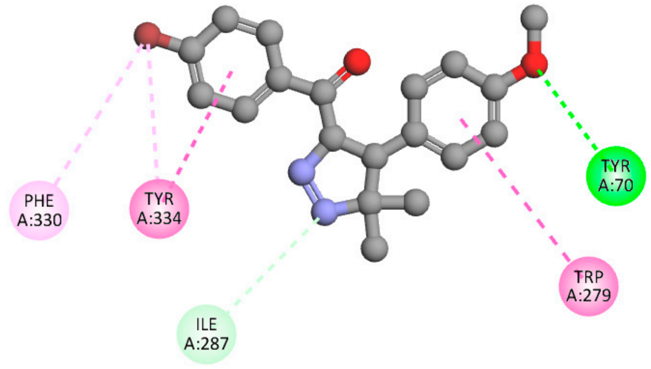

2D depiction of docking of ligand 2 in the active site of AChE

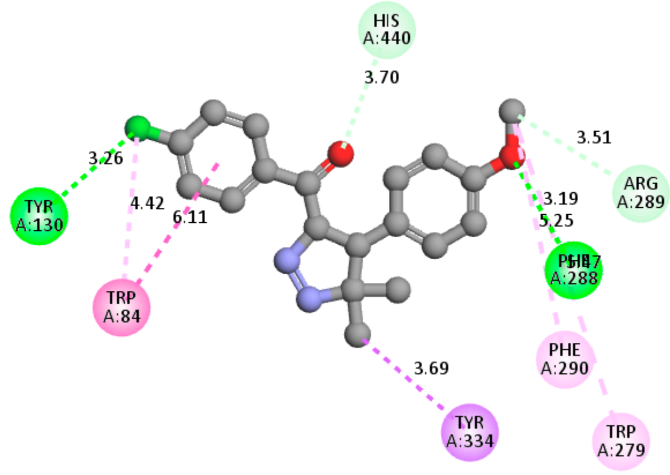

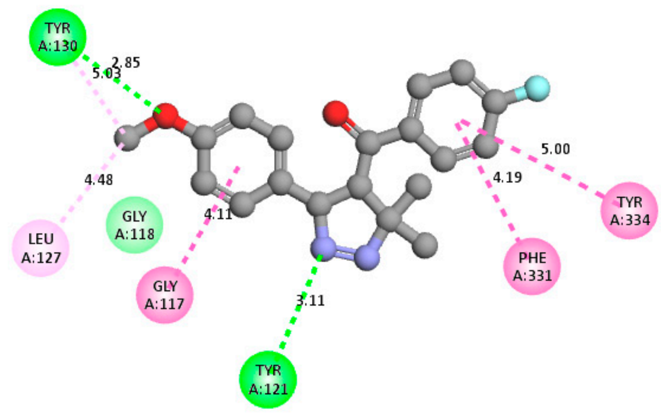

2D depiction of docking of ligand 3 in the active site of AChE

2D depiction of docking of ligand 4 in the active site of AChE

Interactions

Conventioanl Hydrogen Bond | Carbon Hydrogen Bond | Pi-Donor Hydrogen | Halogen (Fluorine)
Alkyl, Pi-Alkyl | Pi-Pi Stacked, Pi-Pi T Shaped, Amide-Pi Stacked | Pi-Cation, Pi-Anion | Pi-Sigma

Figure S16. 2D depiction of docking of ligands 1HP1-4 in the active site of AChE (PDB ID: 1EVE)

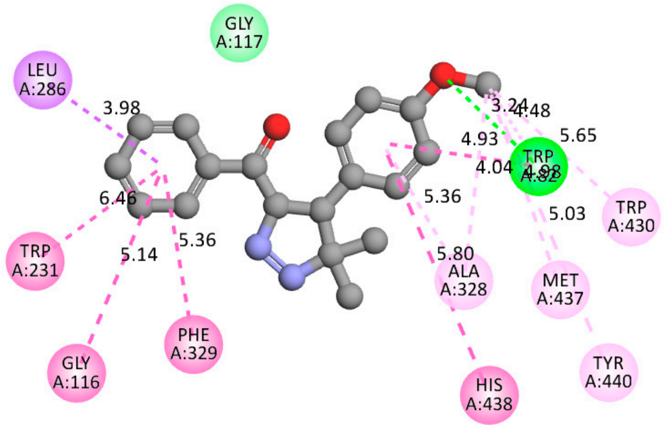

2D depiction of docking of ligand 1 in the active site of BuChE

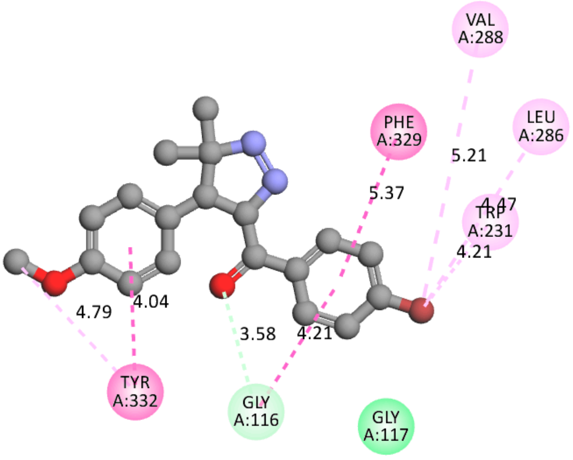

2D depiction of docking of ligand 2 in the active site of BuChE

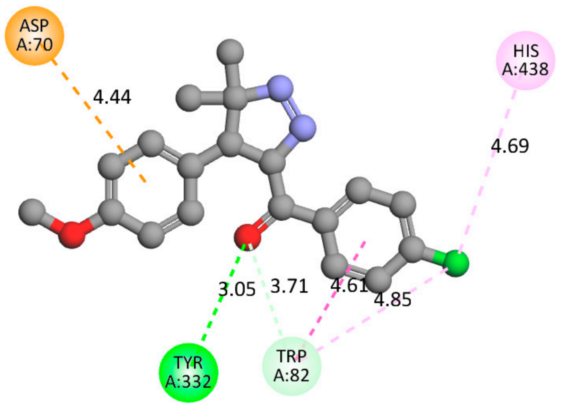

2D depiction of docking of ligand 3 in the active site of BuChE

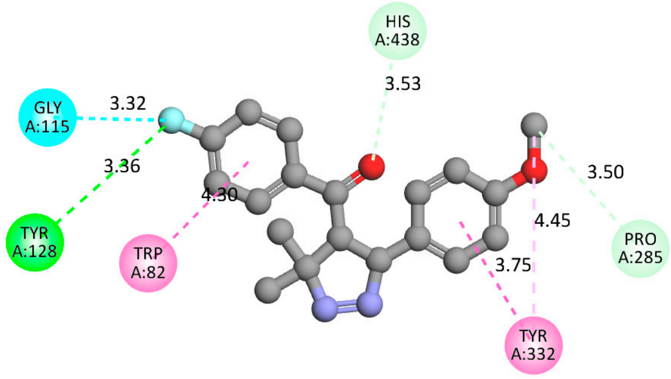

2D depiction of docking of ligand 4 in the active site of BuChE

Interactions

Conventioanl Hydrogen Bond | Carbon Hydrogen Bond | Pi-Donor Hydrogen | Halogen (Fluorine)
Alkyl, Pi-Alkyl | Pi-Pi Stacked, Pi-Pi T Shaped, Amide-Pi Stacked | Pi-Cation, Pi-Anion | Pi-Sigma

Figure S17. 2D depiction of docking of ligands 1HP1-4 in the active site of BuChE (PDB ID: 1P0I)
